# Management and follow-up of uveal effusion syndrome: a case report

**DOI:** 10.1186/s12886-023-03078-x

**Published:** 2023-08-14

**Authors:** Yane Gao, Qiuxin Wu, Jing Xu, Qingmei Tian, Xiaofeng Xie, Xiujuan Du, Hongsheng Bi

**Affiliations:** 1 Shandong Provincial Key Laboratory of Integrated Traditional Chinese and Western Medicine for Prevention and Therapy of Ocular Diseases, Shandong Academy of Eye Disease Prevention and Therapy, Jinan, 250002 Shandong P.R. China; 2 Affiliated Eye Hospital of Shandong University of Traditional Chinese Medicine, Jinan, 250002 Shandong China

**Keywords:** Uveal effusion, Syndrome, Diagnosis, Treatment, Triamcinolone acetonide

## Abstract

**Background:**

We present the management and follow-up of a case of uveal effusion syndrome (UES).

**Case presentation:**

We study the relevant recent literature reports and review the aetiology, clinical classification, pathogenesis, diagnostic characteristics, treatment methods, and prognosis of this disease. When we encounter UES patients clinically, we can classify them according to their clinical characteristics and adopt different treatment plans for different types. The retina of this patient reattached 5 months after receiving eight periocular injections of triamcinolone acetonide (TA).

**Conclusions:**

For type III UES patients, local hormone therapy can be applied, and follow-up should be done to optimize the clinical outcome.

## Backgrounds

Uveal effusion syndrome (UES) is a rare clinical disease. Missed diagnosis and misdiagnosis often occur due to an insufficient understanding of this disease. Treatment of this disease remains challenging. We report one clinical case of UES, study the relevant recent literature reports, and review the aetiology, clinical classification, pathogenesis, diagnostic characteristics, treatment methods, and prognosis of this disease.

## Case presentation

**Fig. 1:**
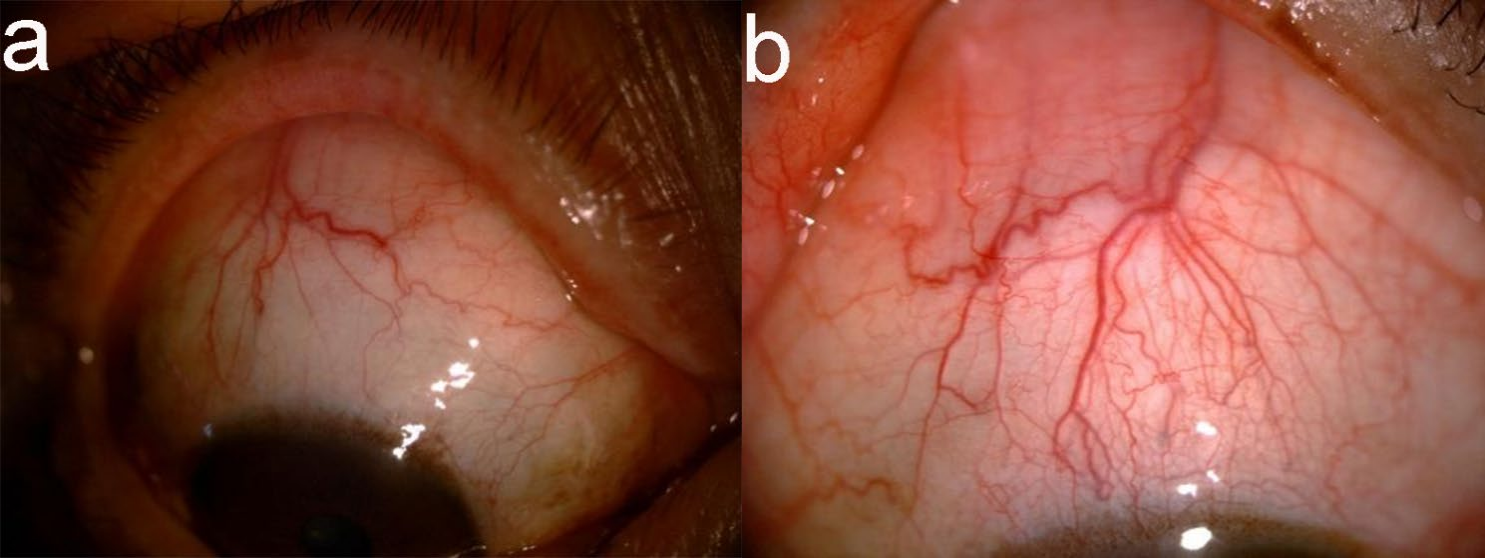
Blood vessels on the scleral surface were tortuous and dilated (**a**: blood vessels on the scleral surface above the right eye; **b**: blood vessels on the scleral surface above the left eye)

**Fig. 2:**
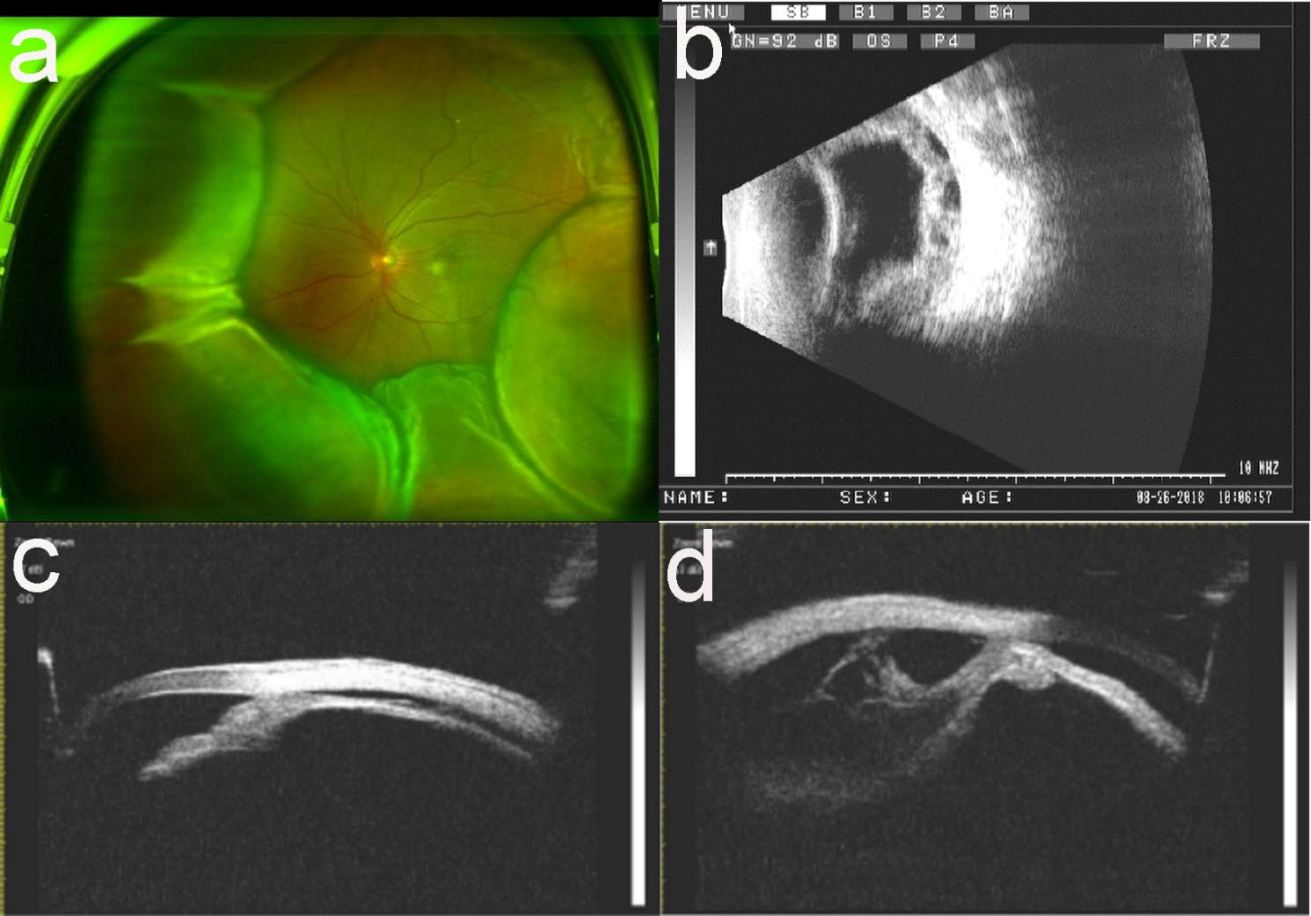
**a**: Anteroposterior view of the left eye fundus. The image shows choroidal detachment and retinal detachment at the peripheral temporal, superior, nasal, and inferior sides. **b**: Left-eye B-ultrasound shows eyeball wall thickening. Coronal scan shows the rosette sign (+). **c**: Right-eye UBM shows the supraciliary effusion without communication with the anterior chamber. **d**: Left-eye UBM shows the supraciliary effusion without communication with the anterior chamber

**Fig. 3:**
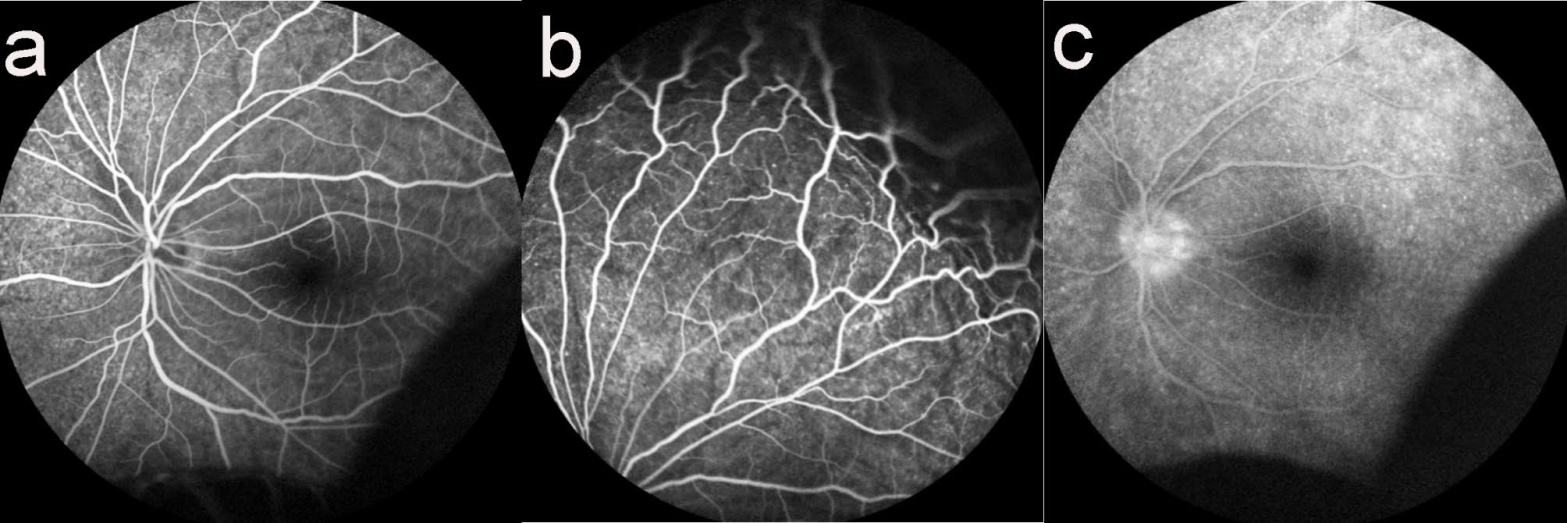
**a**: FFA: There was no delay in arteriovenous filling; fine punctuate hyperfluorescence leakage was observed in the left retina, which gradually enhanced. **b**: Choroidal folds were seen at the posterior pole; peripheral retinal blood vessels were tortuous. **c**: Hyperfluorescence in the optic disc was observed at the late stage, there was diffuse hyperfluorescence under the retina, and fluorescence in peripheral retina was blocked due to choroidal detachment

The patient was 63-year-old man who sought treatment in our hospital due to decreased vision in the left eye for more than 10 d on August 27, 2018. His uncorrected visual acuity was 20/50 in the right eye, which could be corrected to 20/25, and 20/50 in the left eye, which could not be corrected. Slit-lamp examination revealed superficial congestion of the sclera (Fig. 1a), shallow but clear anterior chamber, slight opacification of the lens, and mild vitreal haze in the right eye. Slit-lamp examination of the left eye showed tortuous and dilated blood vessels on the scleral surface (Fig. 1b), shallow anterior chamber with aqueous flare, iris bombe, stellate keratic precipitates (KP), slight opacification of the lens, and mild vitreal haze. Dilated funduscopic examination showed 360° choroidal detachment in the peripheral area, retinal detachment, retinal folds and macular oedema in the left eye but no retinal break (Fig. 2a), and no abnormalities in the right eye. The IOP of the right eye was14mmHg and the left eye was 13.7 mmHg. B-ultrasound scan showed normal right eye and relatively thickened retina and choroid of the left eye. B-ultrasound coronary scanning results showed a rosette sign (+), suggesting retinal detachment and choroidal detachment (Fig. 2b). Ultrasound biomicroscopy (UBM) showed a shallow anterior chamber in both eyes, narrow chamber angle, iris bombe, forward lens location, ciliary process pronation, superciliary body effusion, and no communication with the anterior chamber (Fig. 2c,d). The diagnosis of UES was based on the criteria proposed by Uyama [1]: (1) detection of RD in the lower periphery by fundus examination, without retinal break; (2) presence of subretinal fluid shifting with head position; (3) no leakage from the choroid membrane into the subretinal space revealed by fundus fluorescein angiography (FFA); (4) RD accompanied by peripheral flat or annular ciliochoroidal detachment; (5) detectable ora serrata without the compression of the sclera; (6) exclusion of other causes of ciliochoroidal detachment, including decreased intraocular tension, intraocular tumour, rhegmatogenous retinal detachment, and intraocular inflammation. This patient met criteria 1 and 4, so bilateral uveal effusion syndrome (UES) was preliminarily diagnosed. Further examination showed that the subretinal fluid changed with the patient’s head position, and the subretinal fluid always flowed to the place where the head position was low. Fundus examination after mydriasis showed serrated margins in the superior temporal periphery of the left eye. Optical coherence tomography (OCT) showed that the macular area of the right eye was normal, but retinal diffuse oedema and thickening in the posterior pole of the left eye were observed, combined with effusion under neuroepithelium. FFA showed that the arteriovenous filling time was normal, and fine punctate hyperfluorescence leakage was seen in the retina of the left eye that became gradually enhanced. There was subretinal diffuse hyperfluorescence at the late stage; a choroidal fold was seen in the posterior pole; the peripheral retinal vessels were tortuous; the optic disc had hyperfluorescence at the late stage; the fluorescence was masked in the peripheral retina due to choroidal detachment (Fig. 3a,b,c). Retinal punctate fluorescence leakage was found within approximately 1PD above the nasal side of the right eye and gradually increased. Indocyanine green angiogram showed diffuse hyperfluorescence in the choroid at the early stage that gradually leaked at the late stage. The axial length was 20.62 mm in the right eye and 21.44 mm in the left eye. Orbital magnetic resonance showed no abnormality of the scleral wall.

**Fig. 4:**
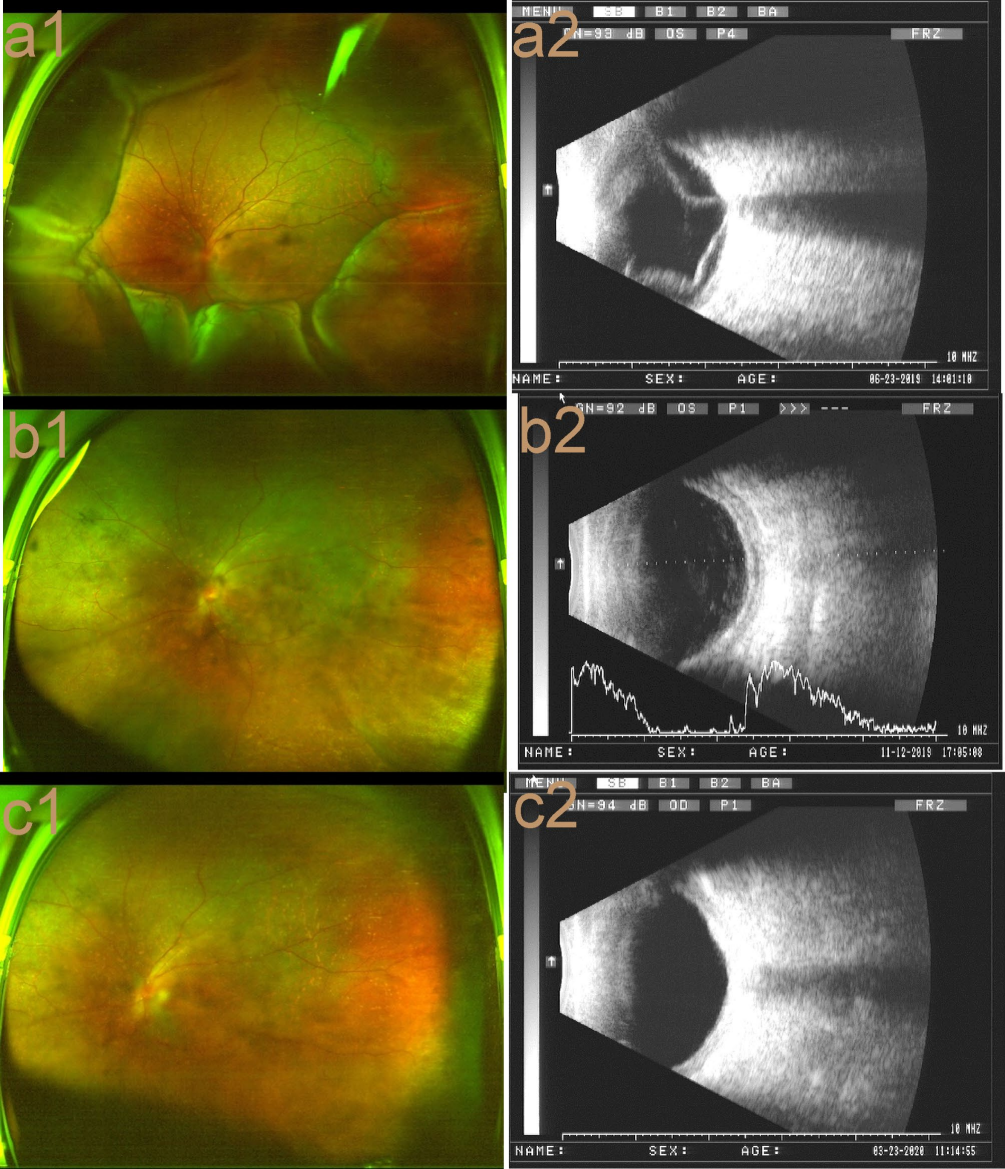
**a1**: Peripheral choroidal detachment and retinal detachment. Many orange-red spots were seen in the posterior pole of the retina. **4a2**: Left-eye B-ultrasound scan showed that there was still eyeball wall thickening. Coronal scan showed the rosette sign (+). **b1**: The left fundus was blocked by posterior lens capsule opacity, the mage was blurry, and many orange-red spots were visible. **4b2**: Left-eye B-ultrasound showed eyeball wall thickening and oedema. There was no choroidal or retinal detachment, and the retina was in place. **c1**: Blurry left fundus. Many orange-red leopard spots were observed in the retina, and the retina was in place. **c2**: B-ultrasound showed that the retina was in place

According to the basic ocular examination, B-ultrasound, UBM, FFA, auxiliary examination by OCT, axial length measurement and orbital MR, the patient was diagnosed with bilateral UES syndrome and bilateral age-related cataract. Some authors [1] classify UES into three types. Given the clinical characteristics of the patient, he was diagnosed with type III UES. Surgery is usually ineffective for type III UES [2]. The effect of oral and peribulbar hormone treatment was better than that of self-healing [3], so the patient was treated with oral prednisone, 70 mg per day. Our planned treatment regimen was 70 mg orally every day for 2 weeks; reducing the dosage by 10 mg per week until it reached 30 mg; then reducing the dosage by 5 mg per week until the dosage reached 15 mg; and taking 15 mg every day for another 6-8months. However, the patient stopped taking the medication himself 2 weeks later on finding no improvement of his left eye. We performed triamcinolone acetonide periocular injections at a dose of 20 mg into his left eye. Our plan was to give 6–8 injections in total, once a month. After the eighth TA injection, the UCVA in the left eye of the patient was 20/100. The posterior lens capsule was slightly opaque, and the fundus was blurred. Peripheral 360° retinal detachment and choroidal detachment were still observed in the fundus, and many orange-red leopard spots were observed in the posterior pole of the retina (Fig. 4a1). B-ultrasound scan showed that the right eye was normal, but the retina and choroid of the left eye were still thickened. Coronal scan by B-mode ultrasound showed the rosette sign (+), suggesting retinal detachment and choroidal detachment (Fig. 4a2).

After that, the patient gave up treatment because he thought it was not effective, and no further medication was given to either eye. Re-examination after 5 months showed that the UCVA in the right eye was 20/200, and the BCVA was 20/20; the UCVA in the left eye was 20/200, which could not be corrected, and the intraocular pressure was 14.0 mmHg. There was posterior capsular lens opacity in the left eye, the retinas and choroid of both eyes were in place (Fig. 4b1). B-ultrasound scan showed that the retina and choroid of the left eye were thickened (Fig. 4b2). Left eye cataract extraction 3 months later was recommended. Because of the COVID-19 pandemic, the patient visited again after 4 months. Fundus photography showed the retina was in place (Fig. 4c1). B-ultrasound scan showed that the left eyeball wall had already returned to normal, and there was no thickening (Fig. 4c2). The preoperative BCVA was 0.04.

**Fig. 5:**
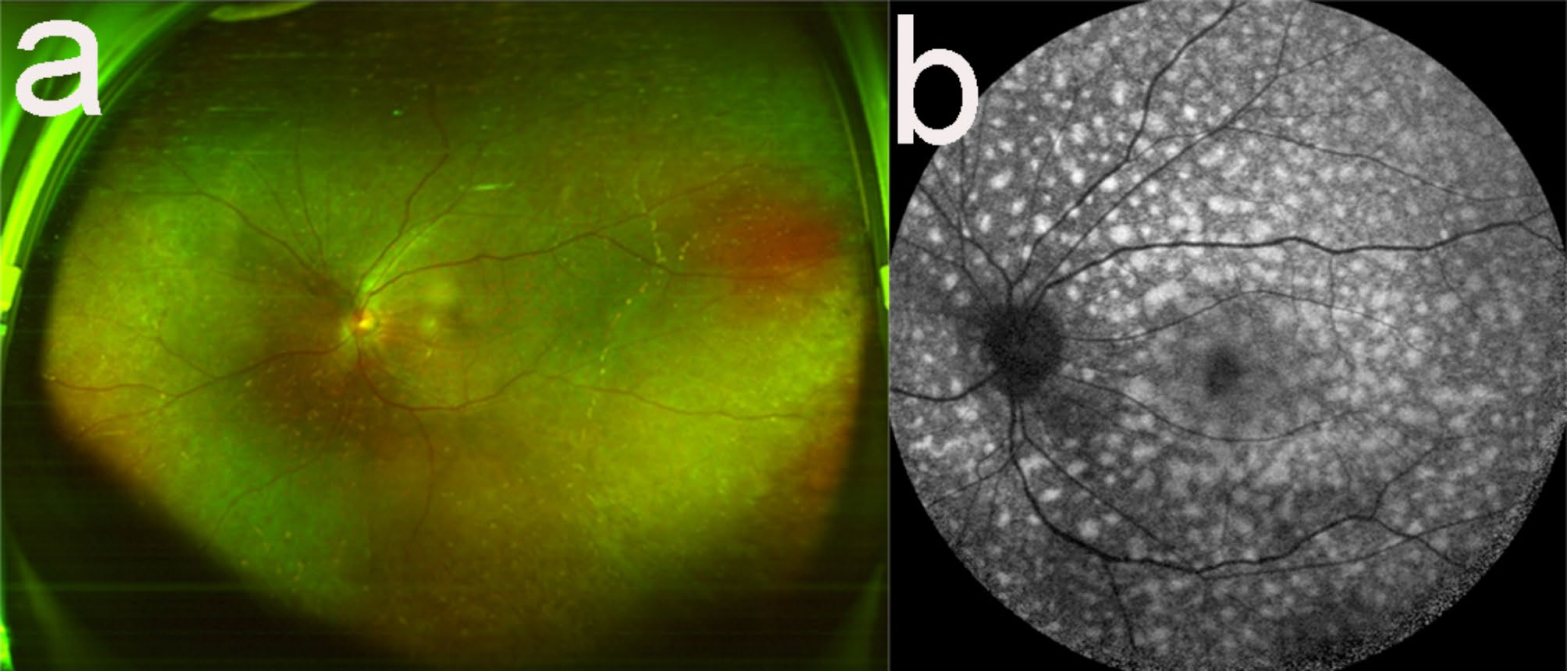
**a**: Colour fundus image at the re-examination 1 year after cataract surgery. Many orange-red spots were observed in the retina. **b**: An autofluorescence image at the re-examination 1 year after surgery. Locations corresponding to orange-red spots indicated high autofluorescence

Left eye phacoemulsification with intraocular lens (PHACO + IOL) implantation was performed. The postoperative BCVA was 20/50.Re-examination 1 year after surgery showed that the left eye BCVA was 20/50. The retina was in place, and many orange leopard spots were observed (Fig. 5a). Fundus autofluorescence images showed many hyperfluorescent dots (Fig. 5b). B-scan results corresponding to the OCT showed hyperpigmentation. The disease of the patient is currently stable.

## Discussion and conclusions

UES is mainly characterized by a series of fundus changes, including ciliochoroidal detachment and serous retinal detachment caused by vorticose vein return disorder and/or choroidal vascular permeability increase [4]. In 1963, Schepens and Brockhurst first described this disease as “uveal effusion” and published a case report [5]. In 1982, Gass et al. called it idiopathic serous detachment of the choroid, ciliary body, and retina, but diseases caused by low intraocular pressure, intraocular inflammation, and intraocular tumour were excluded. It can be classified into two types, idiopathic (normal eyes) and secondary (nanophthalmos) [6]. Uyama et al. first classified this disease into three types. In type I, the patients have nanophthalmic eyes, the average axial length is < 19 mm and is usually 16 mm, and the patients are highly hypermetropic. The sclera is abnormal congenitally. In type II, the eyeball size is normal, the average axial length is > 21 mm, there is no nanophthalmic eye, and there is an abnormal sclera (thickness or component). The sclera usually becomes abnormal, which might be a change secondary to a systemic disease. In type III, the eyeball is normal, the sclera is normal, and there is no eye abnormality, so it is called idiopathic UES [4].

UES is more common in healthy middle-aged people, the majority of patients are male, and it is a chronic disease. The pathogenesis of UES is still not completely clear, though there are several theoretical speculations, including vortex vein occlusion, increased choroidal permeability, choroid intrinsic changes, and decreased scleral permeability [1, 2, 7–11]. The latter is supported by histopathology. Disorder of collagen fibres in the thick sclera and an increase in the amount of mucopolysaccharide reduce the permeability of the sclera to albumin, leading to changes in osmotic gradients. Therefore, fluid is retained in the suprachoroidal and supraciliary spaces [1, 2, 7–11].

Clinical manifestations of UES [12]: The early symptoms are not obvious, there is no red eye or eye swelling, vision does not change or slightly deteriorates, and visual objects have a sense of partial occlusion. At the late stage, retinal detachment involving the macula occurs, and vision deteriorates significantly. The main features include (1) ciliary body and peripheral choroidal detachment. This is the first physical sign of this disease and is also the main basis for its diagnosis. When ciliary body and peripheral choroidal detachment is suspected and fundus manifestation is not obvious, UBM examination can help with the diagnosis. The right eye fundus of this patient did not have obvious abnormality, but UBM showed ciliary body detachment. (2) It is usually accompanied by exudative detachment of the retina and subretinal fluid changes with body position changes. (3) Superficial scleral vessels are usually dilated, but there is no accompanying inflammation of the conjunctiva or anterior segment. (4) It has a chronic disease process. This disease often starts from the periphery. Patients have no subjective symptoms and usually seek treatment due to reduced macular vision caused by the disease. (5) The disease mostly occurs in both eyes at the same time, but sometimes one after the other. Therefore, both eyes need to be examined. (6) FFA shows that the leopard spot–like change is one of the bases of the diagnosis of this disease, but it is not unique to this disease. Leopard spots are usually not present at the early stage but often occur when the disease course is long. This patient did not have leopard spots in the retina at the early stage of diagnosis. After 10 months of treatment, leopard spots were observed in the retina, mostly at or below the posterior pole. FFA showed that their fluorescence was masked. Autofluorescence indicated high autofluorescence. At the same location, OCT showed local retinal pigment epithelium proliferation. (7) The enhanced depth imaging mode of OCT showed thickening of the choroid.

Treatment of UES: Systemic hormone treatment is ineffective for types I and II UES, so surgical treatment is usually performed. Available surgical methods include (a) vortex vein decompression; (b) sclerotomy or resection; (c) subscleral flap sclerectomy; and (d) vitrectomy with intraocular drainage of subretinal effusion combined with equatorial lamellar sclerectomy. The most commonly used method is full-thickness sclerotomy. Surgery is usually ineffective for type III UES. In one study of 59 eyes with type III UES, systemic (oral), local (periocular or topical), or combined use of corticosteroid therapy controlled 95% of cases, and some cases self-resolved without intervention. Only 5% of cases could not be controlled and required surgery [3]. Glucocorticoids are more effective for patients with more obvious features, poorer vision, and more obvious retinal detachment. Cases with no obvious characteristics usually self-resolve without treatment. In another study, the median time to start relieving effusion was 3 months and to complete absorption was 11 months [13]. Mild symptoms naturally subside after several months or 2–3 years; severe symptoms also get naturally absorbed after 2–6 years. However, after retinal detachment was present for several months, atrophy of the inner choroid and retinal pigment epithelium occurred, and salt-and-pepper-like pigment particles were present in the fundus. The central visual acuity depended on the severity of the disease and ranged from several fingers to normal. However, severe cases could lead to complete blindness [4].

The patient was treated with oral glucocorticoid, but it was ineffective, and then local periocular injection of TA was used. After 5 months, the patient’s condition was relieved and the retinal and choroidal detachment recovered (it took 18 months from the visit to recovery). Although pathological examination of sclera was not performed, considering the axial length and the treatment process of the patient, we concluded that the patient had type III UES, namely idiopathic uveal effusion syndrome.

Patients with UES can be classified according to their clinical characteristics, and different schemes, including surgical and non-surgical treatments, can be adopted for different types. Although pathological examination of sclera was not performed, considering the axial length and the results imaging examinations (B-Scan, UBM, CT scan), we concluded that the patient had type III UES, namely idiopathic uveal effusion syndrome. Therefore this patient did not undergo surgery, instead receiving oral prednisone for 2 weeks and 8 monthly periocular injections of TA. Nearly 5 months after the cessation of treatment, the patient’s retina was repositioned. This approach and outcome are basically consistent with earlier findings [4, 13]. The evidence suggests that for patients with type III UES, local hormone treatment can be carried out. If the effect is unsatisfactory, the medication can be stopped and the patient can be observed. Then the disease may relieve itself, which provides a good foundation for clinical treatment.

## Data Availability

The datasets used and/or analysed during the current study available from the corresponding author on reasonable request.
